# Total Laryngectomy: A Review of Surgical Techniques

**DOI:** 10.7759/cureus.18181

**Published:** 2021-09-22

**Authors:** Adit Chotipanich

**Affiliations:** 1 Otolaryngology Department, Chonburi Cancer Hospital, Ministry of Public Health, Chonburi, THA

**Keywords:** total laryngectomy, surgical technique, salvage laryngectomy, voice rehabilitation, neopharynx, pharyngeal fistula

## Abstract

Since the first total laryngectomy was performed in the late 18th century, several improvements and variations in surgical techniques have been proposed for this procedure. The surgical techniques employed in total laryngectomy have not been comprehensively discussed to date. Thus, the main objective of this article was to address controversial aspects related to this procedure and compare different surgical techniques used for a total laryngectomy procedure from the beginning to the end.

Although the management paradigms in laryngeal and hypopharyngeal squamous cell carcinomas have shifted to organ-preserving chemoradiotherapy protocols, total laryngectomy still plays a prominent role in the treatment of advanced and recurrent tumors. The increased incidence of complications associated with salvage total laryngectomy has driven efforts to improve the surgical techniques in various aspects of the operation. Loss of voice and impaired swallowing are the most difficult challenges to be overcome in laryngectomies, and the introduction of tracheoesophageal voice prostheses has made an enormous difference in postoperative rehabilitation and quality of life. Advancements in reconstruction techniques, tumor control, and metastatic management, such as prophylactic neck treatments and paratracheal nodal dissection (PTND), as well as the use of thyroid gland-preserving total laryngectomy in selected patients have all led to the increasing success of modern total laryngectomy. Several conclusions regarding the benchmarking of surgical techniques cannot be drawn. Issues regarding total laryngectomy are still open for discussion, and the technique will continue to require improvement in the near future.

## Introduction and background

The total laryngectomy procedure involves the removal of all laryngeal structures and a section of the upper trachea, which leads to disconnection of the airway and a permanent breathing hole in the neck (tracheostoma). In this approach, a cure for cancers is achieved at the expense of the patient’s voice [1]. Total laryngectomy is also performed for non-cancerous conditions, such as severe trauma or chondronecrosis of the larynx. A reduction in the utilization of total laryngectomy has been observed [2,3] since landmark trials in organ preservation for laryngeal squamous cell carcinoma by the Veterans Affairs Laryngeal Cancer Study Group in 1991 [4], followed by the Groupe d’Etude des Tumeurs de la Tête et du Cou group in 1998 [5] and the Radiation Therapy Oncology Group 91-11 in 2001 [6].

Based on the National Comprehensive Cancer Network Clinical Practice Guidelines in Oncology, total laryngectomy remains the standard treatment for T4a laryngeal squamous cell carcinoma and is a choice besides organ-preserving treatment for T3 laryngeal and T2-T4a hypopharyngeal squamous cell carcinomas [7]. With the exception of tumors with cartilage invasion, patients treated with organ-preserving treatment yield comparable oncologic outcomes to those treated with surgical modalities [8]. Organ-preserving treatment, if successful, preserves nearly normal laryngeal function, resulting in a better quality of life. However, patients with poor general health status or advanced age may not tolerate the toxicity of chemoradiation [4-6]. Organ preservation may not benefit patients with irreversible loss of laryngeal functions [9]. Approximately, one-fourth of patients undergoing organ-preserving treatment required salvage total laryngectomy because of a non-responsive tumor or complication associated with aspiration and necrosis [10]. The advantages and disadvantages of these treatments must be discussed with the patients to allow them to make an informed decision.

Recent decades have shown several changes in operative procedures. The surgical techniques for total laryngectomy have not been comprehensively discussed. This review aims to broadly summarize the variation and improvement in surgical technique for total laryngectomy. Since no previous review has addressed these topics within the scope of a single article, this review should help improve understanding of the surgical technique benchmarking and controversial aspects in each step of the operation.

## Review

Incision

**Figure 1:**
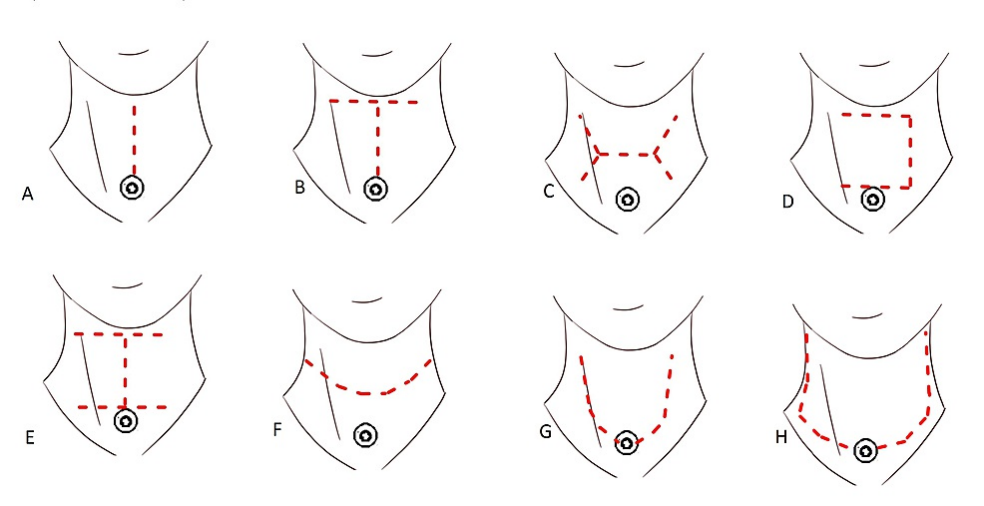
Schematic illustration of the eight different types of neck incisions. (A) Midline vertical incision, (B) T-shaped incision, (C) horizontal double-Y incision, (D) trap-door incision, (E) double trap-doors, (F) apron incision (separate tracheostoma), (G) apron incision (tracheostoma-incorporated), and (H) extended apron incision.

Various incisions have been described for this procedure (Figure 1) [11,12]. A vertical midline incision provides direct access to the larynx but shows limited lateral exposure to the neck. T-shaped and double-Y incisions provide more exposure to the neck, but these incisions involve trifurcation, which results in a poor blood supply and inferior cosmetic results. Trap-door incisions preserve neck tissue for use in multi-stage reconstruction of extensive pharyngeal defects; however, their use has been abandoned in favor of contemporary single-stage reconstructions [12].

Apron incisions are considered the most favorable option for laryngectomy. A short apron incision involves a separate tracheostoma, and a long apron incision incorporates a tracheostoma with the incision. The short apron incision yields the best cosmetic result. The longer skin flap in the long apron incision may show decreased vascularity and increased venous and lymphatic congestion at the distal end. However, the short apron incision also resulted in restricted blood flow at a narrow skin portion between the incision and the tracheostoma.

The long apron incision involves a slightly higher incidence of peristomal dehiscence because the tension in this incision occurs directly to the tracheostoma [11]. The long apron incision provides good exposure to the lower-lateral neck. A larger size tracheostoma can be easily created, with the stoma sited within the main incision [13]. A long apron incision is a default option in patients showing lower-extension tumors or tumors requiring neck dissection.

Management of paratracheal lymph nodes

Paratracheal lymph nodes run along the sides of the trachea. Metastasis to these nodes can lead to peristomal recurrence and poorer clinical outcomes [14,15]. Paratracheal nodal dissection (PTND) may increase the risk of hypocalcemia and is not routinely performed in laryngectomy surgery [16].

Tumors involving the subglottic, pyriform sinus apex, and post-cricoid regions are at risk for paratracheal node metastasis [17,18], and the rate of paratracheal nodal metastasis for tumors in these locations ranges between 12.5% and 51.1% [14-19]. Nodal dissection is the only way to reliably identify occult metastases and extranodal extension, which are crucial in predicting prognosis [20]. Prophylactic PTND shows improved local control and survival rates in salvage laryngectomy [15], but the benefit of adding prophylactic PTND in combined total laryngectomy and radiotherapy remains unclear [19].

Therapeutic PTND is necessary for patients with enlarged paratracheal lymph nodes. Prophylactic PTND should be performed in patients undergoing salvage total laryngectomy for recurrent T3-T4 tumors and is a considerable primary surgical option for tumors involving the subglottic, pyriform apex, and post-cricoid regions. Occult metastasis to contralateral paratracheal nodes is uncommon [18,19], and ipsilateral PTND in non-midline tumors can provide adequate results with minimal morbidity [21]. 

Thyroid gland preservation

The anatomical position of the thyroid gland renders it vulnerable to invasion by advanced laryngeal and hypopharyngeal cancers. In the past, ipsilateral hemithyroidectomy or total thyroidectomy was considered mandatory for all patients undergoing total laryngectomy [22]. However, several patients who underwent laryngectomies with partial preservation of the thyroid gland still developed hypothyroidism because the blood supply to the remnant thyroid gland was compromised during the operation [23]. Moreover, later studies have shown that thyroid gland invasion is uncommon [22-24]. Modern imaging techniques allow more accurate preoperative assessment of thyroid gland invasion. As a result, unnecessary thyroidectomy can be avoided and the incidence of postoperative hypothyroidism can be reduced [25].

Thyroid gland involvement in laryngeal and hypopharyngeal squamous cell carcinomas is mostly caused by direct extension. Lymphatic metastasis to the thyroid gland is rare, but it may occur because of the thyroid gland adjacent to the paratracheal lymph nodes [16-18]. Thyroid or cricoid cartilage invasion detected by preoperative imaging is a strong predictor of thyroid gland invasion [23,25]. Although uncommon, tumors may extend through the cricothyroid membrane or around the thyroid cartilage. These patterns of tumor invasion and lymphatic metastasis to the thyroid gland might not be detectable on CT images [23,25]. Routine thyroidectomy should help prevent missed diagnoses of thyroid gland involvement in tumors with a high risk of occult thyroid gland invasion and lymphatic metastasis.

The amount of thyroid gland tissue that needs to be removed depends on the extent of the tumor. Surgeons should be reminded that a positive surgical margin is associated with a worse prognosis. In cases showing gross invasion of the thyroid gland, total thyroidectomy is not an overtreatment. Hemithyroidectomy and/or isthmectomy are adequate when preoperative imaging shows only cartilage erosion without gross thyroid gland involvement. However, routine thyroidectomy is still recommended for tumors that involve the subglottic, post-cricoid, and pyriform sinus because of the high risk of occult thyroid gland involvement and lymphatic metastasis in these tumors. Apart from these concerns, thyroid-preserving laryngectomy in selected patients does not increase local recurrence rates, nor does it negatively affect disease-free survival [26].

Management of neck metastases

Comprehensive neck dissection (level I-V neck dissection) is generally considered the standard treatment for clinically positive neck nodes (N+) in head and neck cancers [7,27]. In laryngeal and hypopharyngeal squamous cell carcinomas, lymph node involvement is extremely rare at level I (0-2%) and is rare at level V (0-6%), even in cases of clinically N+ [28-31]. A level II-IV select neck dissection (SND) can be alternatively performed in selected cases with limited nodal involvement (N1-N2a) [28].

In patients without clinically detectable lymph node enlargement (N0), prophylactic neck treatment is recommended for any T stage of supraglottic or hypopharyngeal squamous cell carcinoma, and T3/T4 glottic squamous cell carcinoma [7]. Bilateral neck treatment is required if the tumor exceeds the midline or presents with the craniocaudal extension of the larynx [31,32].

Elective neck dissection (END) is recommended as a prophylactic neck treatment in patients for which surgery is used as primary treatment [28,33]. END allows accurate pathological staging and determines whether adjuvant treatment is required [7,34,35]. For example, patients with T3N0 laryngeal squamous cell carcinoma can be safely managed with surgery alone if the pathological results from END show no occult lymph node involvement and other adverse features are not present [36].

Level II-IV SND is commonly used as END in total laryngectomy. Previous studies have shown that in a clinically N0 neck, lymph node level IIB has almost never been involved in supraglottic and glottic squamous cell carcinomas (0-1%), and is rarely involved in hypopharyngeal squamous cell carcinoma (3%) [31,37-40]. For lymph node level IV, occult lymph node involvement is rare to none in supraglottic and glottic squamous cell carcinomas (0-3.5%) [33,41,42]. To decrease postoperative morbidity as much as possible, super-SND at levels IIa and III in supraglottic and glottic squamous cell carcinoma has been advocated by several authors [33,41,42].

Removal of the larynx

After the disinsertion of the strap and suprahyoid muscles and ligation of the feeding vessels, the larynx and trachea are dissected off the constrictor muscles and esophagus. The next crucial step is the removal of the larynx. This can be done using either a closed or an open technique.

The traditional technique requires entering the pharynx. The pharynx can be entered in the following locations: above the hyoid bone, lateral pharyngeal wall, and post-cricoid [43]. The pharynx should be entered at sites away from the tumor. A sufficient surgical margin is necessary for cancer surgery; however, there is no consensus on adequate safety margins in total laryngectomy [44]. In general, a margin of at least 5 mm seems acceptable in laryngeal and hypopharyngeal squamous cell carcinomas [45]. The disadvantages of opening the pharynx are salivary contamination and the need for manual closure.

Reconstruction of the neopharynx

After removal of the larynx, the resulting defect of the pharynx is repaired, creating the so-called neopharynx. The ideal neopharynx must be watertight to avoid leakage, sufficiently large to allow food passage, and capable of accommodating voice rehabilitation.

The neopharynx must retain some degree of pharyngeal function to allow passage of food and production of alaryngeal speech. Loss of elasticity could result in a patulous passage, and conversely, excessive muscle tone can cause spasms; both conditions are undesirable for swallowing and speech rehabilitation [46,47].

In the closed technique, the larynx is separated from the pharynx by using a mechanical stapling device without entering the pharynx [48]. This closed technique significantly shortens the operation time and hospitalization [49]. Post-operative pharyngeal fistula and other complications occurring after stapling closure were shown to be comparable to, if not better than, those occurring after manual closure [48-52]. No study has confirmed the significant benefit of using stapling closure in salvage total laryngectomy [49]. The additional cost of using a stapling device must be considered against the benefits of the reduced operation.

In the closed technique, the lack of visualization of the tumor during resection is associated with the potential risk of inadequate surgical margins. Thus, the use of a stapler for pharyngeal closure should be performed only for tumors confined to the larynx [49,50]. In this regard, it may be safer to use the stapler closure technique for non-cancerous conditions.

Choosing Between Primary Closure or Other Reconstructions

Traditionally, the neopharynx is maintained wide enough to accept a 36-French bougie (approximately 3.8 cm in circumference). Hui et al. demonstrated that the narrowest width of the pharyngeal remnant (about 1.5 cm relaxed or 2.5 cm stretched) is sufficient both for primary closure and for restoration of swallowing function [53]. While these values are the theoretical lower limits of pharyngeal width that can be used in neopharyngeal reconstruction without significant stenosis, a larger neopharyngeal diameter does not correlate with better swallowing outcomes [53,54].

When achievable, primary closure is the first choice for neopharyngeal reconstruction because it has better function and is less complicated [55]. However, when primary closure is not possible, several reconstruction methods can be used. There is an ongoing controversy over the type of reconstruction that offers the best outcome.

In general, the choice of reconstruction is dictated partly by the expertise available and partly by the size of the defect. The commonly used reconstructions are pedicled flaps (e.g., pectoralis major myocutaneous flap) or free vascularized flaps (e.g., free radial forearm flap) in non-circumferential defects and gastric pull-up flaps in circumferential defects [56].

Management of salvage total laryngectomy with sufficient pharyngeal mucosa remains controversial. The fistular rates after salvage laryngectomy with primary closure vary from 10% to 78.6% [57-59]. A pooled analysis showed that primary closure combined with flap reinforcement using vascularized pedicled or free flaps reduced the risk of fistularity by one-third, compared to primary closure alone [60].

However, the routine use of flap reinforcement in salvage total laryngectomy has been a topic of controversy. Despite the scope for extended hospitalization and the potential need for further treatments [61], most fistulae are resolved using only conservative treatments [62]; therefore, routine flap reinforcement may add unnecessary morbidities associated with additional flap harvesting. Thus, vascularized flap reinforcement may be beneficial in patients with considerable post-radiation effects and should be considered on a case-by-case basis.

The Mucosal Layer

**Figure 2:**
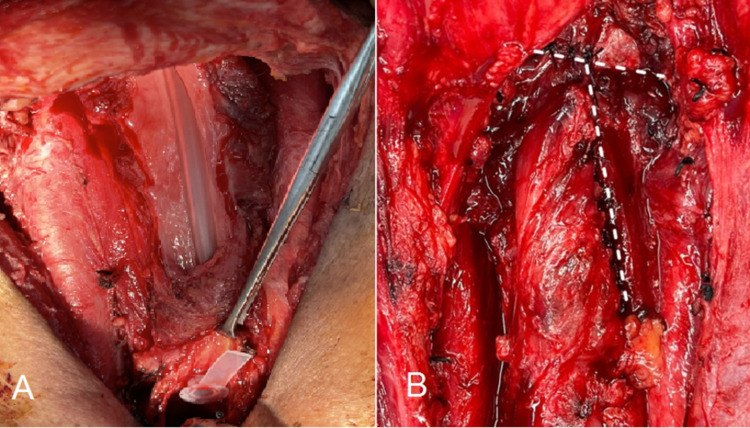
The mucosal layer of the neopharynx. (A) The defect after total laryngectomy with partial hypopharyngectomy and (B) the T-shaped closure.

The mucosal layer of the neopharynx must be repaired to create a watertight closure. The mucosal repair can be either straight (vertical or horizontal) or a T-shaped line. The choice is based on the surgeon’s preference and defect shape. Small defects are usually repaired using straight lines, while in larger defects, mucosal repair often results in a T-shaped line (Figure 2).

Caution should be exercised when applying the vertical line closure since surplus tissue at the midline of the neopharynx can create a pseudo-diverticulum [63], which can cause postoperative dysphagia [64,65]. The pseudo-diverticulum occurs less often when applying the T-shaped closure (84.6% in vertical closure and 18.5% in T-shaped closure) [63]. The swallowing function of horizontal and T-shaped closures was found to be superior to that of vertical closure [63,64].

In theory, the trifurcation in the T-shaped closure might increase the risk of fistula development, which is supported by the findings of some studies [66,67]. However, in contrast, other studies have suggested that a fistula occurs more frequently with vertical closure [63,68,69]. It is assumed that the T-shaped closure causes less tension than the vertical closure in some defect shapes. The horizontal closure appears to be the ideal closure line because it avoids trifurcation and produces a relaxed neopharynx with improved swallowing function [64,70,71]. However, horizontal closure may not be suitable for vertically extended pharyngeal defects.

**Figure 3:**
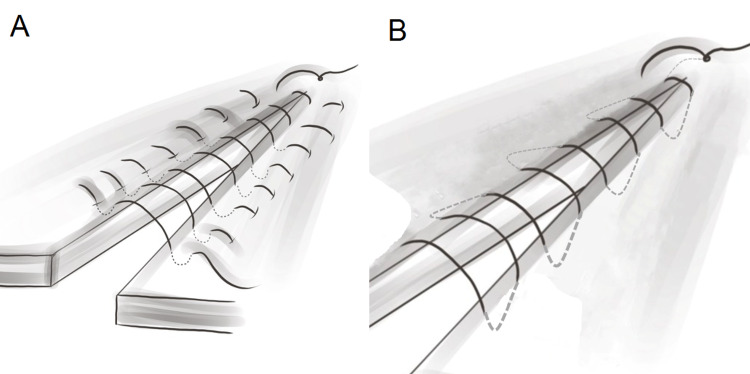
Schematic illustration of commonly used suture patterns in mucosal layer repair. (A) The Lambert and Gambee interrupted-suture techniques and (B) the Cushing and Connell continuous-suture techniques. The Cushing and Lambert techniques penetrate only the submucosa, while the Connell and Gambee techniques pass through the lumen.

The suturing technique for mucosal closure is critical because it must ensure that the mucosa is properly inverted and closed without excessive tension. Numerous techniques are available and can be broadly classified into interrupted and continuous sutures (Figure 3). Continuous sutures have the advantage of evenly distributing the wound tension. However, if a knot breaks, wound dehiscence can occur easily. In this respect, interrupted sutures provide better security. Recent studies have shown that the use of continuous sutures is associated with lower fistula rates than interrupted sutures in mucosal repair [67,72,73].

The Submucosal and Muscular Layers

**Figure 4:**
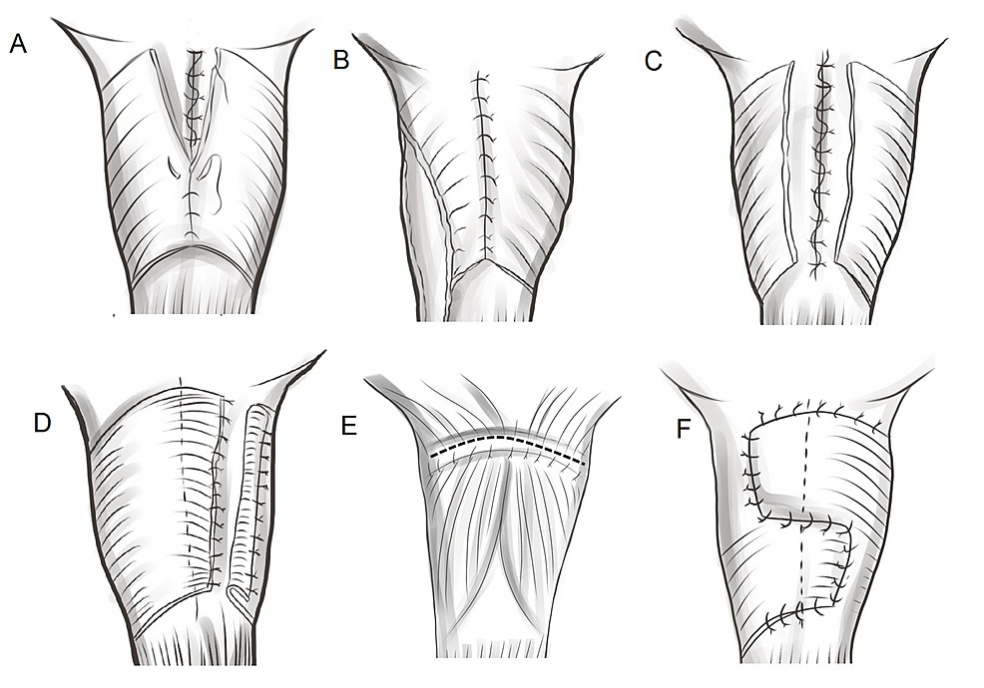
Schematic illustration of the reconstruction of the neopharynx. (A) Traditional three-layer closure, (B) three-layer closure with myotomy, (C) non-closure of the pharyngeal musculature, (D) half-muscle closure technique, (E) horizontal closure, and (F) crossover zigzag neopharyngoplasty. In horizontal closure, the pharyngeal constrictors are stitched to the tongue base muscles.

In the traditional technique, the pharyngeal defect is closed in three layers (mucosal, submucosal, and muscular layers; Figure 4A). However, 12%-35% of patients who underwent the traditional three-layer closure failed to achieve satisfactory speech after tracheoesophageal puncture because of cricopharyngeal spasms [74,75].

The exact mechanism underlying these spasms remains unclear. Surgery may damage the branches of the vagus nerve, resulting in uncoordinated contractions of the pharyngeal constrictor muscles [76]. To avoid spasms, later studies suggested adding either pharyngoesophageal myotomy (Figure 4B) or unilateral pharyngeal plexus neurectomy [77,78]. The advantage of neurectomy is that the vascularity of the pharyngeal wall is not compromised. However, spasms may still occur after the neurectomy because the cricopharyngeal muscle can be innervated from the recurrent laryngeal nerve or the contralateral pharyngeal plexus [79]. Thus, the combination of neurectomy and myotomy may be superior to either technique alone in cricopharyngeal spasm prevention [75]. A study comparing combined myotomy-neurectomy to pharyngeal myotomy alone found a lower incidence of cricopharyngeal spasm in the combined myotomy-neurectomy group, but that study did not show a significant difference in speech outcomes between these two groups [75,80].

Another modified method for preventing cricopharyngeal spasm is to avoid complete circumferential repair of the pharyngeal musculature (Figure 4C-4F) [71]. Non-closure, half-muscle closure, horizontal closure, and crossover zigzag neopharyngoplasty were superior to the traditional three-layer closure. One study also suggests that these modified methods may be performed in combination with pharyngoesophageal myotomy to minimize the intra-luminal pressure of the neopharynx which may lead to an improved voice outcome [80].

Currently, these additional or modified techniques have shown more success in voice restoration than the traditional three-layer closure, and are associated with lower rates of voice restoration failure (between 0% and 10%) [77,79-84]. Based on the literature, pharyngoesophageal myotomy is the most commonly used technique [74-77,79,80,83,84]. However, there is still no consensus on the superiority of any of these additional or modified techniques because of the lack of standard assessments of voice quality and well-controlled studies.

Surgical voice rehabilitation

Currently, there are three methods of voice rehabilitation after total laryngectomy: esophageal speech, electrolarynx, and surgical voice rehabilitation. Esophageal speech is achieved through a process of esophageal insufflation with swallowed air. The air is released from the esophagus, making the juncture of the neopharynx and esophagus a vibratory sound source for alaryngeal speech. Esophageal speech is more difficult to learn than the other two methods [85].

The electrolarynx produces voice using a device to emit constant vibrations that are transmitted to the pharynx through cervical skin or directly to the intraoral mucosa. The electrolarynx does not require surgical intervention or suitable neopharyngeal function. However, current commercially available electrolarynxes provide inferior voice quality compared to the other methods [85].

The idea of surgical voice rehabilitation after total laryngectomy is to create a connection between the trachea and the esophagus, which shunts pulmonary air to the neopharynx and esophagus juncture. The tracheoesophageal connection needs to be stable enough to prevent spontaneous closure while simultaneously allowing air to be easily drawn into the esophagus and preventing the aspiration of saliva [86].

Previously, tracheoesophageal connections were created using the patient’s own tissues. Various techniques with moderate success rates have been described for this purpose, but all of them had high complication rates, mainly due to shunt breakdown and aspiration. Because of these and the complexity of some of the procedures, these techniques have been abandoned since a tracheoesophageal voice prosthesis (TEP) was invented [87].

Currently, TEP is considered the gold standard for voice rehabilitation. TEP uses a one-way valve to push air up from the lungs to pass through from the trachea and enter the esophagus without letting food or liquids pass through the other way. Patients simply occlude a stoma with a finger or a hand-free valve. The surgical process is simple, with the only insertion of a TEP through a tracheoesophageal puncture behind the tracheostoma. A tracheoesophageal puncture can be performed at the time of laryngectomy (primary puncture) or at a later date (secondary puncture) [88].

During larynx removal, care should be taken to keep the tracheoesophageal party wall intact at the puncture site. Separation of this wall could cause a postoperative fistula. Primary TEP is considered to be associated with an increased risk of surgical complications, such as infection, stoma stenosis, fistula, and leakage [89,90]. However, these complications are infrequent and usually not severe. Moreover, there is no robust evidence to suggest that primary TEP is associated with poorer outcomes than secondary TEP [89].

Apart from the reasons already mentioned, primary TEP should be preferred because it avoids a second surgical intervention and allows early voice restoration, thereby exerting a positive psychological impact on patients who have undergone total laryngectomy.

Tracheostoma creation

The final step of the operation is the creation of a tracheostoma. The skin and the tracheal opening are sutured together using half-mattress sutures, which pull the skin over the exposed trachea ring. The diameter of the stoma should be more than 14 mm to ensure an adequate airway [91]. However, a stoma that is too large makes speech using stoma occlusion difficult; thus, the stoma should not be larger than the patient’s thumb.

Stenosis of post-laryngectomy tracheostoma is common. The stomal construction is an important determinant of stomal stenosis. Constricting scars and stenoses occur when the trachea is insufficiently anchored to the skin and when raw areas are left at the junction between the skin and the tracheal mucosa. Diabetes mellitus and related tracheostoma infection are also considered risk factors for tracheostoma stenosis [92].

**Figure 5:**
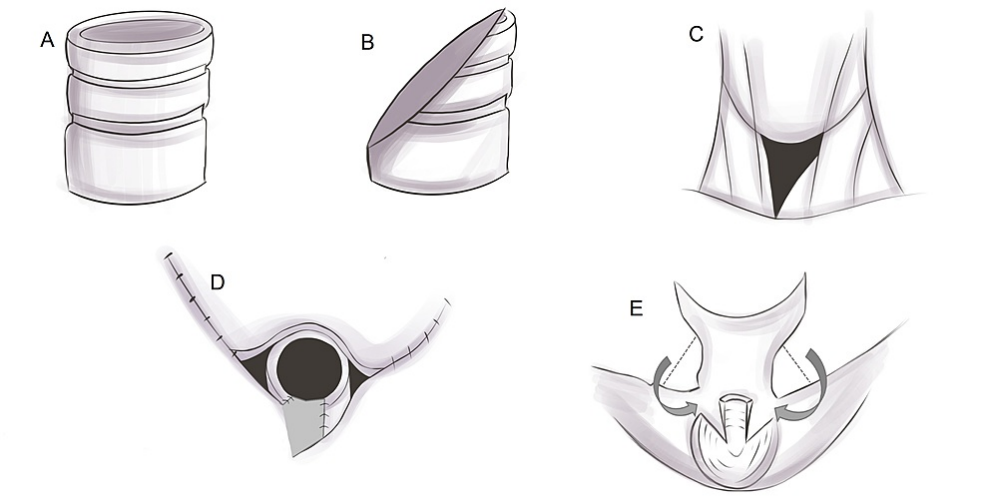
Schematic illustration of different stoma-fashioning techniques. (A) Straight transection, (B) beveled transection, (C) wide triangular stoma, (D) interposed lower skin flap, and (E) Y-shaped, interposed superior skin flap.

A simple technique to avoid tracheostoma stenosis is to create a larger stoma. A straight transection of the trachea (Figure 5A) results in a smaller diameter and a higher stenosis rate compared to the beveled technique (Figure 5B) [93]. The stoma can be created with a much greater diameter when the tracheal opening is extended to the lower neck by four or five tracheal rings, forming a triangular shape (Figure 5C) [94]. The triangular stoma technique fully prevents stenosis, but its size and shape are troublesome for speech using stoma occlusion.

Another technique is the use of a skin flap interposed in the tracheal opening to prevent circular scars [93,95]. This technique, which can be performed with various designs (Figure 5D-5E), can effectively prevent stenosis while maintaining proper stoma shape.

The use of heat-and-moisture-exchange filters and hands-free speech valves requires a shallow and flat peristomal area for proper fitting of the adhesive patches [96]. An excessive stomal depth is undesirable for tracheoesophageal speech and tracheostoma care. Thus, division of the sternal heads of the sternocleidomastoid muscle is usually performed during a total laryngectomy to prevent recession and shrinkage of the tracheostoma [97].

## Conclusions

Surgical techniques used in total laryngectomy are improving gradually. To remain relevant, several changes in operative procedures have been introduced to reduce complications and improve quality of life without compromising tumor control. These changes are partly driven by the increased complications of salvage total laryngectomy in the era of organ-preserving treatment. Moreover, the introduction of surgical voice rehabilitation can provide reasonable functional restoration and show a significant positive impact on the patient’s quality of life. These are important issues that have recently gained attention.

Although many changes in total laryngectomy have been observed in recent decades, several conclusions related to benchmarking for surgical techniques cannot be reached. This situation can be primarily attributed to the lack of adequate and well-controlled studies, necessitating further studies. Thus, although total laryngectomy is one of the oldest cancer surgeries, issues related to the procedure remain open for discussion and will continue to require improvements in the near future.
